# Dip-Coating of a Nanometer-Thick Fluorinated Imidazolium Ionic Liquid: Layer Structure, Formation Mechanism and Wetting Behavior

**DOI:** 10.1021/acs.langmuir.6c00733

**Published:** 2026-04-07

**Authors:** Abdulmalik Dahiru, Lei Li

**Affiliations:** Department of Chemical and Petroleum Engineering, 6614 University of Pittsburgh, Pittsburgh, Pennsylvania 15260, United States

## Abstract

In this work, the film formation mechanisms and wetting behavior have been investigated for a nanometer-thick highly fluorinated imidazolium-based ionic liquid (HFILOH) deposited on silica substrate via dip-coating. Through systematic variation of solution concentration and dwell time, it is found that the nanometer-thick HFILOH forms a dual-layer structure consisting of a self-limiting bonded layer and a concentration-dependent mobile layer. Kinetic modeling reveals that the formation of the bonded layer follows reversible pseudo-first-order adsorption kinetics, with the equilibrium bonded thickness remaining constant at ∼0.8 nm across all concentrations, while characteristic time constants decrease from ∼2080 s to ∼ 314 s with increasing concentration. The mobile layer thickness exhibits excellent linear correlation with concentration (*R* ^2^ = 0.99), consistent with Landau–Levich hydrodynamic theory for dilute solutions. These results demonstrate that HFILOH film formation in dip-coating is governed by the dual mechanisms of site-limited adsorption and viscous flow entrainment. Contact angle measurements reveal hydrophilic-oleophobic wetting behavior, with hexadecane contact angles remaining consistently high (∼70–75°) while water contact angles increase modestly from ∼ 30° to ∼ 40° with dwell time, indicating time-dependent molecular reorganization and preferential orientation of fluorinated chains at the HFILOH-air interface. This interpretation of a fluorine-rich outer surface is in good agreement with angle-resolved X-ray photoelectron spectroscopy and dynamic contact angle measurements previously reported for nanometer-thick HFILOH coatings on silica, which directly show fluorinated segments enriched at the film–air interface and stable hydrophilic–oleophobic wetting. Together, these findings provide mechanistic insight into the structure–property relationships controlling ionic liquid nanofilm functionality and establish HFILOH as a promising candidate for applications like antifogging, self-cleaning, and oil–water separation.

## Graphical abstract

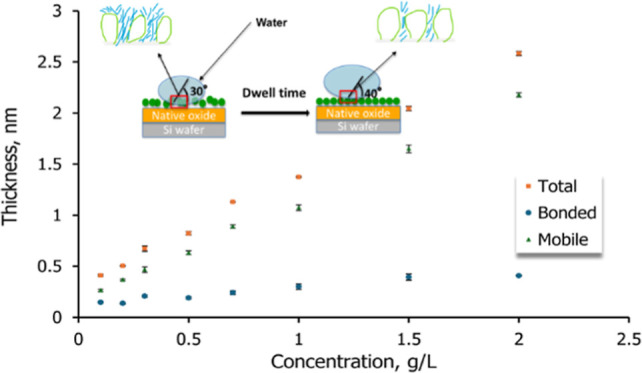

## 1 Introduction

Ionic liquids (ILs) have emerged as a versatile class of materials distinguished by their nonvolatile nature, high thermal stability, and molecular tunability, which have enabled their use in energy, catalysis, and surface modification.^1,2^ Among these, imidazolium-based ILs are particularly notable because their cation and anion structures can be systematically varied to tune properties such as viscosity, thermal and electrochemical stability, and ionic conductivity.^2^ In addition, studies on alkyl and fluoroalkyl imidazolium ionic liquids show that structural modifications strongly influence interfacial composition and ordering at liquid–vapor interfaces, highlighting the tunability of their surface behavior.^3^ The application of these compounds in thin film coatings is an area of rapidly growing interest, since surface-engineered IL films can provide precise control over surface wettability and interfacial behavior.^4,5^ Dip-coating is a particularly attractive, scalable process for ionic liquid coatings because it delivers uniform films and adjustable thicknesses at nanoscale, all of which have been demonstrated on silica and other oxide surfaces.^6^ Indeed, dip-coating is the state-of-the-art process to apply nanometer-thick lubricant on the magnetic media for hard disc drive (HDD) industry.^7^ In dip-coating, the substrate is immersed in a coating solution and then withdrawn at a controlled speed. During withdrawal, a liquid film is entrained on the surface and subsequently thins by drainage and solvent evaporation, leading to the formation of the coating. The final film structure and thickness depend on the interplay between hydrodynamic entrainment, governed by viscous flow and described by Landau–Levich theory, and surface adsorption processes, which can create strongly anchored molecular layers.^6,7^ For functionalized lubricants such as perfluoropolyethers (PFPEs), this dual mechanism produces a dual-layer structure: a bonded layer formed by adsorption of end groups to surface sites, and a mobile layer controlled by hydrodynamic film entrainment.^7^ Understanding how these two mechanisms operate for ionic liquids is essential for controlling film structure and properties.

A major challenge is to understand and control the mechanisms of ionic liquid nanofilm formation, especially for highly fluorinated imidazolium species with advanced surface properties and selective wetting behavior.^8^ Molecular packing, fluorination, and substrate chemistry have been shown to strongly affect film morphology and wettability for these ILs, as tailored fluoroalkyl groups enrich at the interface and produce surfaces with low surface tension, robust antifouling, and resistance to hydrocarbons.^3,9^ These selective interfacial properties are commonly attributed to nanoscale organization in ionic liquid films, where ionic and fluorinated segments segregate into structured domains with small space between chains. In such architectures, small polar molecules like water can access residual polar sites, whereas larger nonpolar hydrocarbons primarily encounter low-energy fluorinated groups and are effectively excluded.^28^ This ability to design and control wettability and adhesion through molecular structure is leading to next-generation antifogging, self-cleaning, and oil–water separation coatings based on tailored fluorinated ionic liquids.^8,9^

Despite progress, systematic exploration of deposition-structure–property relationships is still needed to unlock the full potential of novel ionic liquids for advanced coatings. In particular, the kinetics of film formation, the relative contributions of adsorption versus hydrodynamic processes, and the time-dependent evolution of film structure remain poorly understood for fluorinated imidazolium ionic liquids. While PFPE lubricants have been extensively characterized in terms of bonded and mobile layer formation during dip-coating,^7^ comparable studies for ionic liquids are lacking. Although the dual hydrophilic-oleophobic character of fluorinated imidazolium films has been demonstrated,^10^ the connection between dip-coating process parameters, film formation kinetics, and the resulting wetting behavior has not been established.

This work presents a detailed study of film formation mechanisms and wetting behavior for a newly developed fluorinated imidazolium-based ionic liquid, HFILOH, deposited on silica substrates via dip-coating. The objectives of this study are 2-fold: (1) to uncover the underlying mechanisms governing the structure of the nanometer-thick HFILOH in dip-coating process. (2) to investigate the time-dependent wetting properties and their relationship to molecular reorganization of nanometer-thick HFILOH. By employing dip-coating on silica substrates and characterizing layer formation and wetting property, this study aims to provide new insight into film growth mechanisms and tunable wettability. The experimental results show that HFILOH forms a dual-layer structure analogous to PFPE lubricants, with an adsorption-limited bonded layer and a concentration-dependent mobile layer controlled by Landau–Levich hydrodynamics. The deposition mechanism, together with the intrinsic hydrophilic–oleophobic character of fluorinated imidazolium films, enables control of film structure while preserving dual wetting functionality. The study suggests that ionic liquids are promising as next-generation coating materials, not only as lubricants but also as multifunctional wetting modifiers. In particular, Wang et al. (2022) reported that HFILOH can function as a next-generation media lubricant for hard disk drives, underscoring its potential for advanced tribological applications.^9^

## 2 Experimental Methods

### 2.1 Materials

The synthesis of HFILOH (highly fluorinated imidazolium ionic liquid with hydroxyl functionality) has been described previously in our laboratory.^9^ The reactants were 1-iodo-1H,1H,2H,2H-perfluorohexane (99%; TCI America), 1-(2-hydroxyethyl)­imidazole (97%; Sigma-Aldrich), and lithium bis­(nonafluorobutanesulfonyl)­imide (95%; TCI America). Acetone (propan-2-one, ≥99.5%, Sigma-Aldrich), isopropanol (propan-2-ol, ≥99.5%, Fisher Scientific), ethyl acetate (HPLC grade, Fisher Chemical), diethyl ether (99.5%, extra dry, stabilized, AcroSeal packaging; ACROS Organics), and Vertrel XF (fluorinated hydrofluoroether solvent, Miller-Stephenson Chemical Company) were used as received. Deionized (DI) water was produced using a Millipore Academic A10 system (total organic carbon <40 ppb). Silicon substrates consisted of silicon wafers with a 2 nm native oxide layer (P/B ⟨100⟩, 1–10 Ω·cm, 279 ± 25 μm) purchased from Silicon Quest International, Inc. For film preparation, HFILOH solutions were diluted by dissolving the ionic liquid in Vertrel XF, in which it readily dissolves, to obtain coating solutions with concentrations ranging from 0.1 to 2.0 g/L.

### 2.2 Substrate Preparation

Silicon wafers were used as substrates. Prior to dip-coating, wafers were sequentially rinsed in acetone, isopropanol (IPA), and Vertrel XF, each for 2 min, followed by oven drying at 70 °C for 5 min. The substrates were then treated with UV/ozone for 5 min using a BioForce Nanosciences ProCleaner (110 V AC, 50/60 Hz, 0.5 A, single phase; 185 and 254 nm UV wavelengths). This dual cleaning protocol (solvent washing + UV/ozone) was carried out in ambient air at room temperature to remove organic residues and activate the native oxide surface.

### 2.3 Dip-Coating Procedure

Thin films of HFILOH were deposited using a KSV Instruments dip coater. Substrates were vertically immersed in HFILOH/Vertrel XF solutions at varying concentrations (0.1–2.0 g/L), held for the desired dwell times, and then withdrawn at a constant speed of 1 mm/s. After withdrawal, the solvent evaporated under ambient conditions, leaving behind an ionic liquid nanofilm. Washed films were prepared by subjecting freshly coated substrates to two sequential dips in pure Vertrel XF at the same withdrawal speed. This step removed mobile HFILOH, leaving only the strongly adsorbed fraction, i.e., bonded fraction, on the surface.

### 2.4 Thickness Measurements

Film thicknesses were determined using a J.A. Woollam Alpha-SE spectroscopic ellipsometer at an incident angle of 70° and beam diameter of ∼2 mm, over a wavelength range of 380–900 nm.The optical constants of the native oxide layer were first determined using the “NTVE_JAW” refractive index database after UV/ozone treatment but before dip coating. After coating, the Cauchy dispersion model was applied to fit the film thickness, using

1 $$n\left(\lambda \right)=A+\frac{B}{\lambda^{2}}$$

where n is the refractive index, λ is the wavelength in micrometers, and constants were set to *A* = 1.45 and *B* = 0.0025 for HFILOH.^28^ Each thickness value represents the average of at least three repeats per sample at different locations, and all reported data are based on ≥ 3 independent samples.

### 2.5 Contact Angle Measurements

The static hexadecane contact angle (HCA) and water contact angle (WCA) of HFILOH-coated films were measured with a VCA Optima contact angle system. Test drops (1 μL) were dispensed automatically, and droplet profiles were recorded using a CCD camera. Contact angles were determined using a vendor-supplied image analysis software. For each condition, at least three droplets were measured on different positions of the substrate, and the reported values represent the average.

## 3 Results & Discussions

### 3.1 Concentration Dependence of Total, Bonded, and Mobile Thickness

Figure 1 shows the variation of total, bonded, and mobile thicknesses of HFILOH films with solution concentration at a fixed dwell time of 30 s and a withdrawal speed of 1 mm s^–1^. As the HFILOH concentration increases from 0.1 to 2.0 g/L, the total film thickness rises monotonically. The bonded thickness gradually increases and then levels off to a plateau of approximately 0.4–0.5 nm. The mobile fraction continues to grow with concentration and accounts for most of the variation in total film thickness.

**Figure 1:**
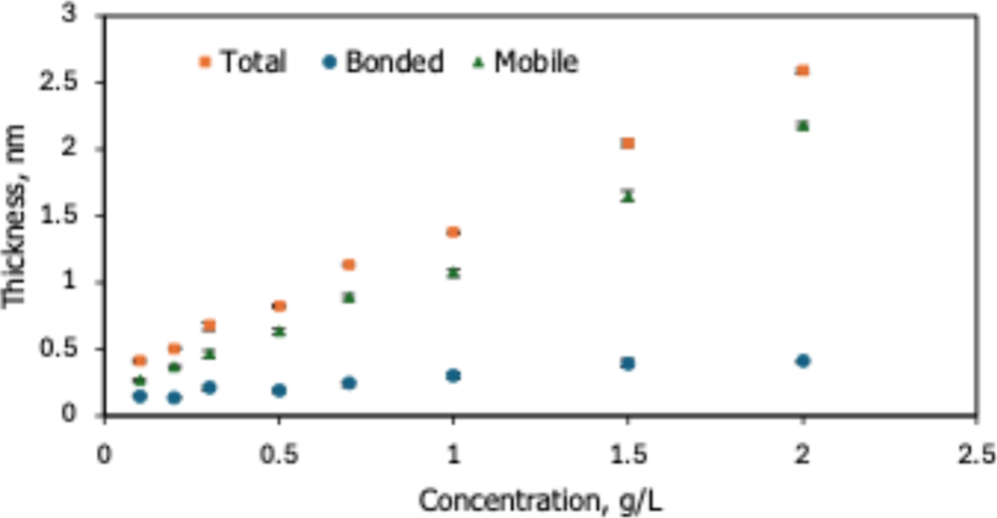
Dependence of total, bonded, and mobile thicknesses of HFILOH films on coating-bath concentration at a dwell time of 30 s and withdrawal speed of 1 mm s^–1^.

### 3.2 Effect of Dwell Time on Total and Bonded Thickness

Figure 2 shows dwell-time-dependent thickness for 0.5, 1.0, and 1.5 g/L solutions. Across all concentrations, the total thickness initially increases with dwell time and then gradually levels off.

**Figure 2:**
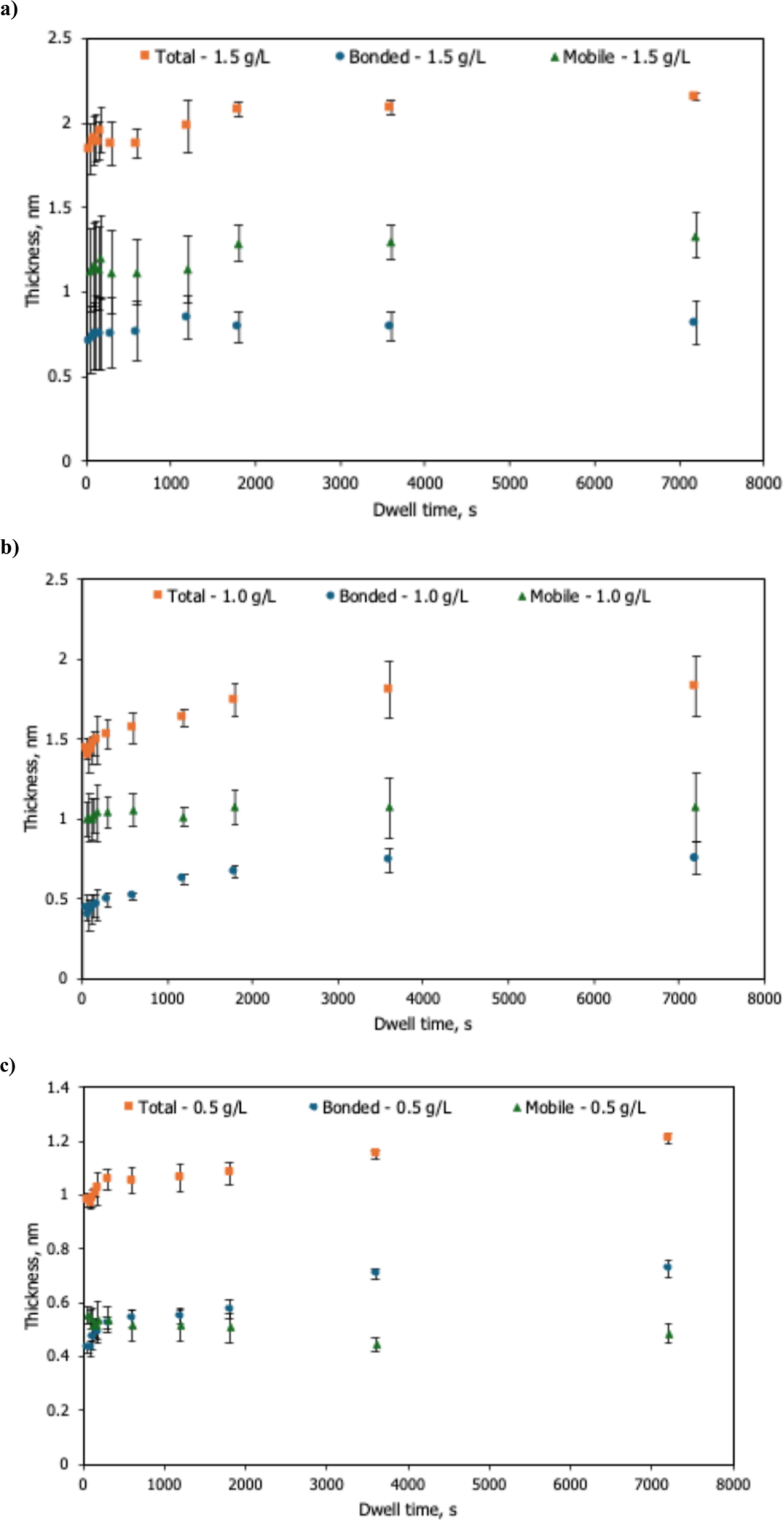
Dwell-time dependence of total, bonded, and mobile thicknesses of HFILOH films at concentrations of (a) 1.5 g L^–1^, (b) 1.0 g L^–1^, and (c) 0.5 g L^–1^ (withdrawal speed = 1 mm s^–1^).

The bonded thickness increases with dwell time before leveling off at longer immersion. The plateaued bonded layer thickness is ∼0.5–0.8 nm across all concentrations. The mobile thickness remains relatively steady at each concentration (∼0.5 nm at 0.5 g/L, ∼ 1 nm at 1.0 g/L, and ∼ 1.2 nm at 1.5 g/L).

### 3.3 Contact Angle Behavior

Figure 3 shows the water and hexadecane contact angles (WCA and HCA) for HFILOH coatings as a function of dwell time and concentration. Across all samples, the hexadecane contact angle (HCA) remains relatively constant at around 70–75°, while the water contact angle (WCA) increases modestly from approximately 30° at short dwell times to around 40° at longer times before plateauing. Bonded-only films (after rinsing) exhibit HCA of about 60–64° and WCA ≈30–35°.

**Figure 3:**
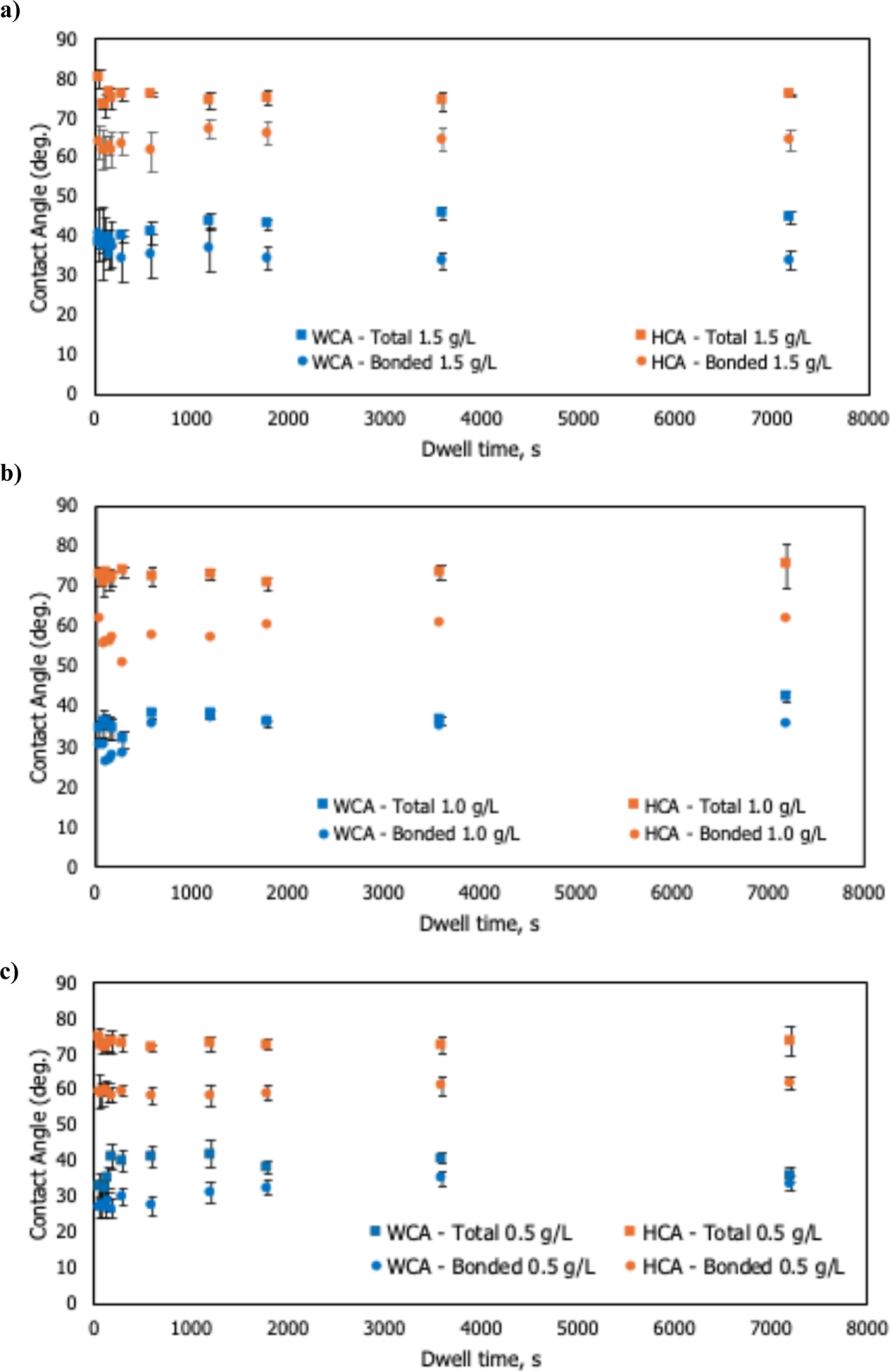
Water (WCA) and hexadecane (HCA) contact angles of HFILOH coatings as a function of dwell time at (a) 1.5 g L^–1^, (b) 1.0 g L^–1^, and (c) 0.5 g L^–1^.

### 3.4 Dual-Layer Structure and Formation Mechanisms

The observed concentration-dependent behavior (Figure 1) reveals two distinct deposition regimes. The bonded fraction represents a strongly anchored layer. This behavior, where a stable, subnanometer anchored layer plateaus at constant thickness independent of solution concentration, is established for ionic liquid films and can be described by adsorption-limited kinetics,^11^ while highly ordered monolayers or checkerboard structures, anchored by a combination of H-bonding and electrostatic interaction, are commonly observed for ionic liquids on oxide and metal surfaces.^12,13^ This site-controlled adsorption mechanism can also describe HFILOH films, where hydroxyl and imidazolium groups interact with surface hydroxyls through hydrogen bonding and electrostatic attraction.

In contrast, the mobile fraction increases approximately linearly with HFILOH concentration, indicating that more solute is retained in the film at higher bath concentrations. This behavior is consistent with a picture in which the hydrodynamic carrier film described by Landau–Levich model remains nearly unchanged at the fixed withdrawal speed used here, and the observed growth of the mobile thickness reflects the higher amount of HFILOH present within that carrier film as concentration increases.^6,14^ Taken together, the concentration-independent plateau in bonded thickness and the dwell-time-independent mobile fraction that increases linearly with solution concentration at fixed withdrawal speed are consistent with a dual-layer structure comprising a site-limited bonded layer and a concentration-dependent mobile layer governed by Landau–Levich-type hydrodynamic entrainment. This decoupled behavior of a self-limiting anchored layer and a tunable mobile layer is characteristic of the HFILOH system studied here and is consistent with adsorption-controlled ionic liquid films discussed in recent interfacial studies.^11^

This decoupling between adsorption and hydrodynamics agrees with previous dip-coating models developed for PFPE lubricants and related systems.^6,7^

The gradual leveling of total thickness with dwell time (Figure 2) is consistent with the combined contributions of hydrodynamic entrainment and interface adsorption. Similar time-dependent growth and eventual saturation of ionic liquid films at solid interfaces have been reported in ultrahigh-vacuum studies on metal surfaces.^12,13^ The bonded thickness behavior suggests that most HFILOH molecules capable of surface attachment anchor within the early stages of immersion, after which adsorption sites become saturated. Such interfacial saturation is characteristic of reversible site-limited adsorption kinetics, as described for dip-coated PFPE films by Merzlikine et al. (2004),^7^ who reported saturation times on the order of 10^3^–10^4^ s for hydroxyl-terminated PFPEs (Z-dol, AM2001:8 × 10^–4^ M; Z-tetrol: 5.6 × 10^–4^ M) under dilute conditions; in contrast, both bonded and total HFILOH film thicknesses in this work reach a plateau within about 1.8 × 10^3^–3.6 × 10^3^ s over 0.5–1.5 g/L, indicating comparable or shorter adsorption time scales under the studied conditions. Experimental and modeling studies confirm that surface-active ionic liquids can form stable, nanometer-scale anchored layers following swift site saturation.^9^ The plateaued bonded layer thickness (∼0.5–0.8 nm) across all concentrations and time scales highlights the finite density of accessible surface-active sites and is characteristic of adsorption-limited regimes.^7,12^ The steady mobile thickness at each concentration is analogous to the dwell-time-independent mobile PFPE layers reported by Merzlikine et al.^7^

### 3.5 Kinetic Modeling of Bonded-Layer Formation

To describe HFILOH adsorption kinetics quantitatively, bonded thickness data were modeled using a reversible pseudo-first-order framework, as validated for dip-coated organic monolayers^7^ and adsorption processes across organic and ionic systems.^15^ This approach is used for monolayer formation at solid/liquid interfaces, including PFPE lubricants on silica.^7^

$${HFILOH}_{\left(sol\right)}+S_{\left(surface\right)}⇌{HFILOH-S}_{\left(bonded\right)}$$

Assuming first-order adsorption and desorption, the rate expression is

2 $$\frac{{dh}_{B}}{dt}=k_{1}C_{A}\left(h_{eq}-h_{B}\right)-k_{2}h_{B}$$

Where *k*_1_ and *k*_2_ are the forward (adsorption) and backward (desorption) rate constants. Such rate equations are appropriate for both physical and chemisorption systems when mass transfer and the density of available sites control the approach to equilibrium.

Integration yields the solution

3 $$h_{B}\left(t\right)=y_{0}+A_{0}\left[1-e^{-t/\tau }\right],\tau =\frac{1}{k_{1}C_{A}+k_{2}}$$

The resulting fits (Figure 4) show excellent agreement with experiment (*R* ^2^ > 0.9), confirming that HFILOH adsorption follows first-order reversible kinetics. As summarized in Table 1, the characteristic time constant, τ, decreases sharply with increasing concentration, from about 2080 s at 0.5 g L^–1^ to ≈314 s at 1.5 g L^–1^, indicating that adsorption accelerates at higher concentration. The fits to the bonded HFILOH layers yield a positive initial thickness y_0_ (Table 1), interpreted as the instantaneous adsorption offset from rapid initial anchoring during immersion. Meanwhile, the equilibrium bonded thickness, *h*_eq,_ remains nearly constant (∼0.8 nm), indicating a site-saturated monolayer independent of solution concentration. This self-limiting behavior matches the adsorption-controlled regime reported by Merzlikine et al. in PFPE study.^7^ The linearized relationship

4 $$\frac{1}{\tau }=k_{1,eff}+k_{2}$$

was used to evaluate the kinetic constants. Following established statistical analysis protocols for adsorption kinetics, both unweighted ordinary least-squares (OLS) and weighted least-squares (WLS) regressions were performed to assess the influence of uncertainty in τ.

**Figure 4:**
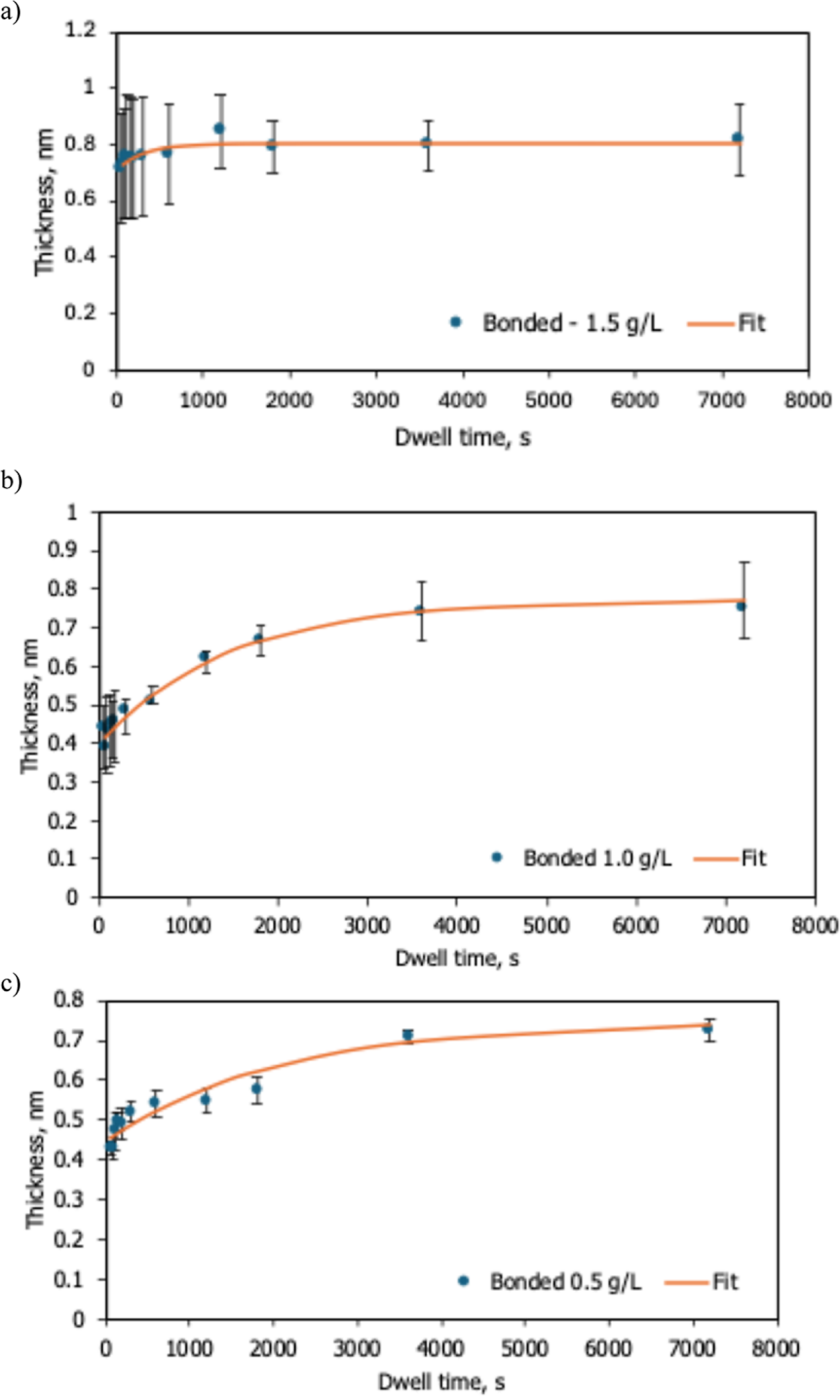
Time-dependent bonded-layer growth of HFILOH films at concentrations of (a) 1.5 g L^–1^, (b) 1.0 g L^–1^, and (c) 0.5 g L^–1^ fitted to the reversible-adsorption model *h*_B_(*t*) = *y*_0_+*A*_0_[1-e^‑*t*/*τ*^].

**Table 1:** Fitted Parameters for Bonded Film Growth *h*_B_(*t*) = *y*_0_+*A*_0_[1-e^‑*t*/*τ*^of HFILOH Coatings at Different Concentrations^a^

concentration (g/L)	*y*_0_ (nm)	*A*_0_ (nm)	τ (s)	*h*_B,eq_ = *y*_0_ + A_0_ (nm)	*R* ^2^
0.5	0.445	0.301	2080.701	0.746	0.927
1.0	0.400	0.372	1451.202	0.773	0.984
1.5	0.709	0.097	314.139	0.805	0.757

a y_0_: instantaneous bonded offset, *A*_0_: amplitude of bonded growth, τ: characteristics time constant, *R* ^2^: goodness of exponential fit.

The unweighted OLS fit yielded *k*_1,eff_ = 2.7 × 10^–3^(1/(s.g L^–1^)) and *k*_2_ = −1.3 × 10^–3^s^–1^ (R^2^ = 0.81) (Table 2), capturing the general trend but producing an unphysical negative intercept. When experimental errors were incorporated through WLS regression, the results became both statistically and physically consistent, giving *k*_1,eff_ = 4.3 × 10^–4^(1/(s.g L^–1^)) and *k*_2_ = 2.6 × 10^–4^s^–1^ (*R* ^2^ = 0.95). This process of kinetic parameter extraction, especially the distinction between unweighted and weighted fits, follows modern recommendations for linearized adsorption analysis.^16–18^ Thus, the data fit the adsorption model well while indicating that HFILOH adsorption is dominated by the forward process (*k*_1_C), with desorption (*k*_2_) being small but finite. The contrast between unweighted and weighted fits underscores the importance of accounting for heteroscedasticity in experimental kinetics: when variance is considered properly, the extracted parameters align with the expected physical picture of near-irreversible bonding. The overall analysis confirms that HFILOH adsorption on silica is dominated by nearly irreversible surface binding with minimal desorption, a behavior in agreement with contemporary adsorption models for ionic liquids and other monolayer-forming adsorbates.^11,12^

**Table 2:** Linearization Kinetic Parameters From 1/τ = *k*_1,eff_C + *k*_2_ Regression

fit type	*k*_1,eff_ (1/(s.g L^–1^))	*k*_2_ (1/s)	*R* ^2^
unweighted	0.00273	–0.00127	0.81
weighted	0.00043	0.00026	0.95

The Landau–Levich (L–L) model provides a classical framework for describing dip-coated films.^6,19^ For a Newtonian liquid of viscosity η, surface tensionγ_LV_, and density ρ, withdrawn from a bath at velocity *U*_0_, the film (including solute and solvent) thickness is given as

5 $$h_{0}=0.94\frac{{\left(\eta U_{0}\right)}^{2/3}}{\gamma_{LV}^{1/6}{\left(\rho g\right)}^{1/2}}$$

Since all experiments in this study were conducted at a fixed withdrawal speed of 1 mm s^–1^ and the HFILOH solutions were highly dilute in Vertrel XF, both η and γ_LV_ can be considered constant. Under these conditions, the hydrodynamic film height *h*_0_ remain effectively constant, regardless of changes in solution concentration. Consequently, any observed variation in mobile-layer thickness is attributed to variations in solute (HFILOH) retained within the constant hydrodynamic film following drainage and evaporation, consistent with classical and contemporary dip-coating theory for dilute solutions.^20–22^ In this scenario, the retained HFILOH molecules constitute the “mobile” fraction, while the bonded layer arises via specific molecular adsorption.

The excellent linear correlation (*R* ^2^ ≈ 0.99, Figure 5) between mobile-layer thickness and concentration demonstrates that in the dilute limit, film buildup is dominated by solute retention rather than changes in hydrodynamic behavior, a result solidly predicted by Landau Levich theory and widely confirmed in dip-coating literature.^23,24^

**Figure 5:**
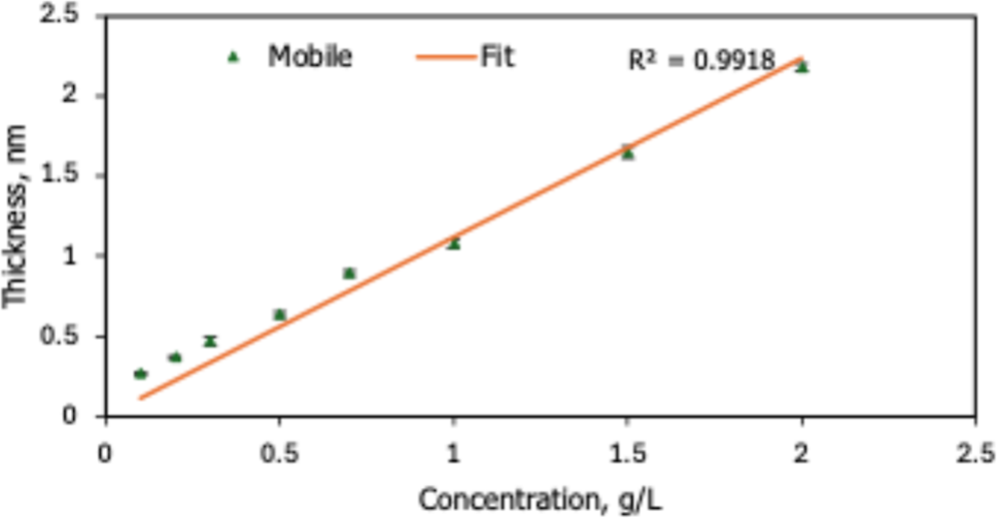
Linear increase of mobile-layer thickness with HFILOH concentration (*R* ^2^ = 0.9918) at a fixed withdrawal speed of 1 mm s^–1^, confirming solute-controlled retention within a constant Landau–Levich film.

### 3.6 Wetting Properties and Molecular Reorganization

The consistently high HCA values (Figure 3) indicate an oleophobic surface driven by outward-oriented fluorinated moieties, as seen even under partial surface coverage, which is supported by observations of high oil contact angles (HCA >70°) in imidazolium-based ionic liquids with fluorinated tails on silicon oxide and other metal oxide substrates.^10^ This immediate oleophobicity is attributed to the preferential orientation of low-energy fluorinated groups at the interface upon adsorption, a phenomenon repeatedly described in fluorinated ionic liquids and polymeric coatings.^25,26^ Angle-resolved X-ray photoelectron spectroscopy on nanometer-thick HFILOH coatings on silica by Tirado et al. further supports this interpretation: for monolayer-thick films, the ratio of fluorinated C 1s peaks to N 1s increases at a 45° incident angle relative to 0°, indicating that fluorinated carbon chains preferentially occupy the coating–air interface while the imidazolium cation and sulfonimide anion reside deeper in the film.^27^

In contrast, the gradual rise in WCA (Figure 3) signals time-dependent molecular reorganization (Figure 6). The fact that the HCA remains high (∼70–75°) while the WCA increases from ∼ 30° to ∼ 40° with dwell time indicates that the surface is fluorine-rich from the outset, sufficient to repel hexadecane, whereas additional adsorption and lateral rearrangement mainly reduce the exposure of polar hydroxyl/imidazolium groups to water. At short dwell times, incomplete or disordered adsorption exposes polar hydroxyl sites, resulting in a more hydrophilic surface with low WCA.^10^ As dwell time increases, continued adsorption and lateral rearrangement favor fluorinated chain orientation toward the air interface, partly screening polar groups and thus increasing the WCA. A similar time-dependent molecular reorganization has been demonstrated for IL films, including AFM-observed transitions from metastable 3D droplets to stable 2D bilayers on AU(111).^12,28^ The modest WCA increase (30→40°), therefore, signals the transition from a polar-exposed interface to a more ordered fluorine-terminated surface.

**Figure 6:**
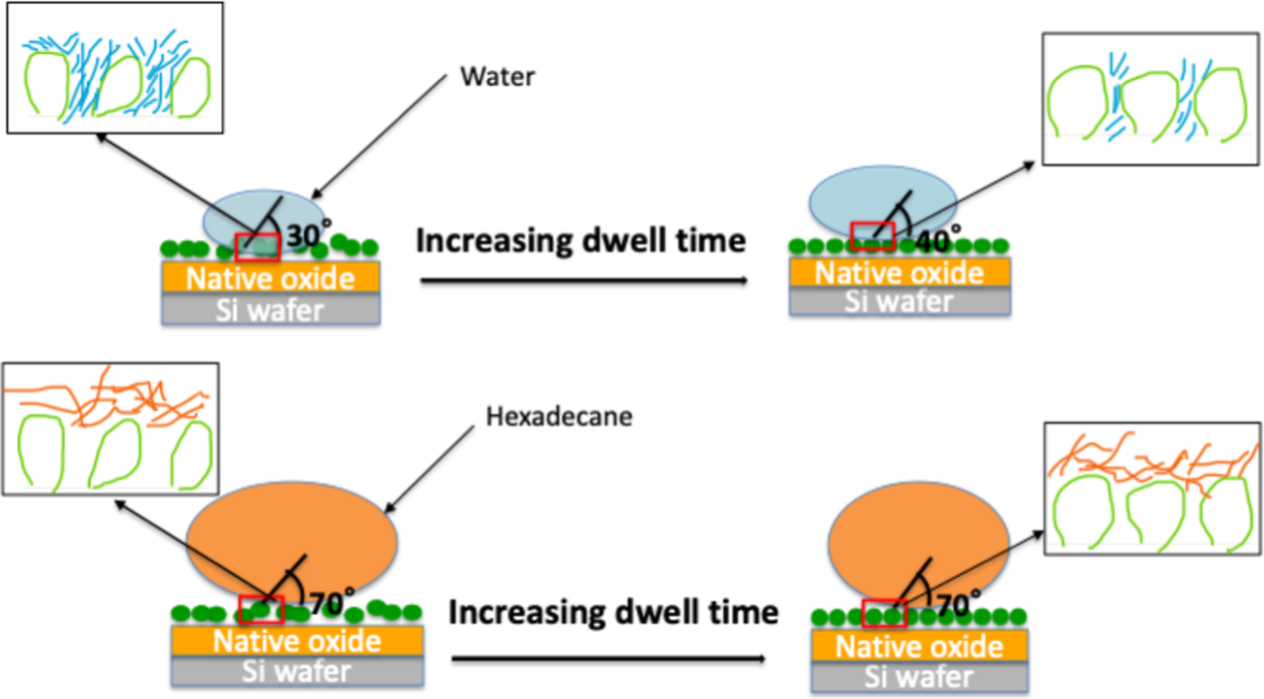
Schematic illustration of the molecular orientation and wetting evolution of HFILOH films with increasing dwell time.

Nevertheless, even at long dwell times, the WCA does not approach the HCA, indicating that residual polar sites remain partially accessible and preserve significant hydrophilicity (θ_water_ < θ_hexadecane_). This polar–nonpolar selective wetting is a hallmark of fluorinated ionic liquid films and has been exploited for oil–water separation and antifogging applications.^10,29^ Selective penetration of small water molecules into nanoscale gaps between tilted ionic groups has also been identified as a mechanism for maintaining hydrophilicity alongside oleophobicity.^29^

Moreover, the overall hydrophilic–oleophobic regime we observe (moderate water contact angles with hexadecane contact angles around 70–75°) is in very good agreement with the static and dynamic contact angles reported by Tirado et al. for nanometer-thick HFILOH coatings on silica and glass, where low-hysteresis hexadecane wetting and stable hydrophilic–oleophobic behavior were observed.^27^ Small, polar water molecules can partially penetrate the nanoscale spacing between tilted ionic groups, whereas nonpolar hexadecane, with a larger molecular size and lacking specific interactions, experiences strong repellence from the fluorinated outer layer.

## 4 Conclusion

In the current work, the film formation mechanisms and wetting behavior have been studied for a nanometer-thick fluorinated imidazolium-based ionic liquid (HFILOH) deposited via dip-coating on silica substrates. Through systematic experiments varying concentration and dwell time, it was demonstrated that the nanometer-thick HFILOH forms a dual-layer structure consisting of a self-limiting bonded layer and a mobile layer, analogous to the nanometer-thick PFPE lubricant. Kinetic modeling revealed that the formation of the bonded layer follows reversible pseudo-first-order adsorption kinetics, with characteristic time constants decreasing from ∼ 2080 s to ∼ 314 s as concentration increases from 0.5 to 1.5 g/L. The mobile layer thickness exhibits excellent linear scaling with concentration (*R* ^2^ = 0.99), consistent with Landau–Levich hydrodynamic theory for dilute solutions. Wetting analysis revealed the unique hydrophilic-oleophobic behavior of HFILOH films, driven by time-dependent molecular reorganization and preferential fluorinated chain orientation at the film–air interface. The hexadecane contact angle remains consistently high (∼70–75°), while the water contact angle gradually increases from ∼ 30° to ∼ 40° with dwell time, indicating progressive ordering of the fluorinated surface layer while preserving access to polar functionalities. These wetting trends are in very good agreement with the static and dynamic contact angles reported by Tirado et al. for nanometer-thick HFILOH coatings on silica, and with their angle-resolved X-ray photoelectron spectroscopy results showing fluorinated segments enriched at the coating–air interface, which together provide independent support for a fluorine-rich outer surface on HFILOH nanofilms. The combination of site-limited bonding with Landau–Levich-controlled mobile layer formation enables straightforward control of film structure through simple adjustment of concentration and dwell time. The mechanistic insights obtained here, together with previously demonstrated long-term hydrophilic–oleophobic performance, antifogging, and detergent-free cleaning behavior of HFILOH coatings, establish HFILOH as a promising candidate for applications in antifogging, self-cleaning, and oil–water separation, and other surface engineering applications.
